# A combined automatic organ at risk contouring and synthetic Computed Tomography solution for pelvic Magnetic Resonance-only radiotherapy

**DOI:** 10.1016/j.phro.2026.100918

**Published:** 2026-02-09

**Authors:** Jonathan J. Wyatt, Sandeep Kaushik, Cristina Cozzini, Bernadett Kolozsvári, Borbála Deák-Karancsi, Rachel A. Pearson, Steven Petit, Marta Capala, Juan A. Hernandez-Tamames, Katalin Hideghéty, Ross J. Maxwell, László Ruskó, Florian Wiesinger, Hazel M. McCallum

**Affiliations:** a Translational and Clinical Research Institute, Newcastle University, Newcastle, UK; b Northern Centre for Cancer Care, Newcastle upon Tyne Hospitals NHS Foundation Trust, Newcastle, UK; c GE Healthcare, Munich, Germany; d Department of Quantitative Biomedicine, University of Zurich, Zurich, Switzerland; e GE Healthcare, Budapest, Hungary; f Erasmus MC Cancer Institute, University Medical Center Rotterdam, Department of Radiotherapy, The Netherlands; g Department of Radiology and Nuclear Medicine, Erasmus MC, Rotterdam, The Netherlands; h Department of Oncotherapy, University of Szeged, Szeged, Hungary

**Keywords:** MR-only radiotherapy, Magnetic Resonance, Synthetic Computed Tomography, deep learning, Pelvic cancers, Automatic Contouring

## Abstract

**Background and Purpose:**

Magnetic Resonance (MR)-only radiotherapy improves treatment accuracy and efficiency but requires a synthetic Computed Tomography (sCT) for dose calculations. Automatic MR-based Organs At Risk (OARs) contouring could further improve consistency and efficiency. A combined automatic MR OAR contouring and sCT solution has been developed, with each component separately validated. This study aimed to evaluate this combined MR-only workflow against a manual MR-CT workflow for pelvic cancers.

**Materials and Methods:**

Radiotherapy MR and CT scans were acquired for 20 patients (10 prostate, 4 rectum, 6 anus cancer). The MR-only workflow used sCTs and automatic contours for the bladder, bowel bag, femoral heads, rectum, penile bulb, prostate, seminal vesicles and urethra along with manual target contours to optimise a MR-only plan. Doses were compared to the current clinical standard of deformably registered CT and manual MR OAR contours (MR-CT).

**Results:**

Mean dose differences were ≤1Gy; 1 percentage point relative volume for all OARs except the bowel bag. Mean absolute dose differences were also ≤1Gy for all OARs except bowel bag in ano-rectal patients and except bowel bag, bladder V50Gy, V40Gy, V30Gy and rectum V30Gy in prostate patients.

**Conclusions:**

The automatic MR OAR contouring and sCT workflow had similar dose differences to manual inter-observer variability for all OARs except the lower dose bladder and rectum volumes (prostate patients only) and bowel bag (prostate and ano-rectal patients). This combined OAR contouring and MR-only workflow could enable more efficient and consistent pelvic radiotherapy treatment.

## Highlights

• Ano-rectal patients: dose differences ≤ 1 Gy for all organs except bowel bag.
• Prostate patients: differences ≤ 1 Gy for femoral heads, penile bulb and urethra.
• These differences are similar to manual inter-observer variability.
• Automatic workflow may perform similarly to manual contouring for these organs.

## Graphical abstract

**Figure:**
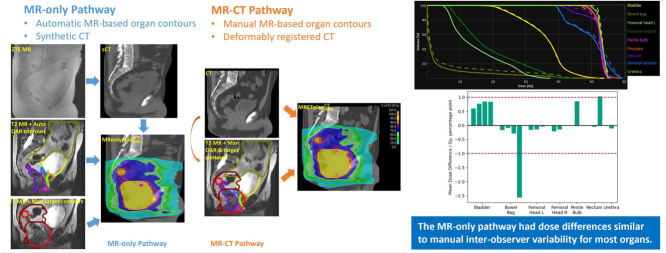

## 1 Introduction

Magnetic Resonance (MR) is the preferred imaging modality for radiotherapy contouring for pelvic cancers due to its superior soft-tissue contrast compared to Computed Tomography (CT) [1], [2], [3]. The improved image quality has motivated the development of MR-based automatic organ at risk (OAR) contouring algorithms, which can further improve efficiency and consistency in contouring [4], [5].

MR-based deep learning models for OAR contours in prostate radiotherapy have demonstrated good concordance with manual contours, with Dice coefficients in the range 0.69 to 0.93 [6] and 0.67 to 0.93 (excluding urethra) [4]. Deep learning MR-based models have also shown high Dice coefficient values for bladder and femoral heads in rectum radiotherapy patients (all 0.93) [7]. However, Dice coefficients have very limited correlation with clinically relevant differences between contours [8] and to our knowledge MR-based pelvic OAR contours have not been evaluated using dose differences previously.

The superior soft-tissue contrast of MR has also motivated MR-only radiotherapy, which can improve treatment efficiency by omitting a planning CT appointment and treatment accuracy by removing the MR-CT registration uncertainty [9]. Radiotherapy dose calculations cannot be performed directly on MR images, but methods of generating a synthetic (s)CT from MR images have also been developed [10]. There have been a large number of sCT algorithms evaluated for pelvic radiotherapy [11], with a number of commercial solutions now available using deep learning models [12]. Most sCT algorithms have demonstrated high dose accuracy, with mean dose differences ≤1% [13]. There is also some evidence that MR-only radiotherapy leads to reductions in target volumes and consequently lower doses to OARs [2], [14].

Combining MR-based automatic contouring with MR-only radiotherapy would enable further improvements in treatment efficiency and treatment accuracy. This could enable radiotherapy departments to continue delivering high quality radiotherapy despite the rising cancer incidence rate [15]. However, to our knowledge, a combined MR-based automatic contour and MR-only radiotherapy workflow has not been evaluated in the literature previously.

A combined automatic MR OAR contouring and sCT solution for the pelvis has been developed, with each component separately validated. Clinicians rated 81% of automatic MR OAR contours as clinically acceptable [4] and mean PTV dose differences of the sCT to CT were ≤0.5% [16]. The aim of this study was to evaluate the combined automatic MR OAR contouring sCT workflow, without manual editing, against the current clinical standard of manual MR contours on a MR-CT workflow for radiotherapy for cancers of the anus, prostate and rectum.

## 2 Materials and methods

### 2.1 Patients and treatment characteristics

A prospective study was performed to evaluate the combination of the deep learning automatic OAR contouring and sCT generation algorithms. The study included 20 patients planned for radical/neoadjuvant radiotherapy for anal (n = 6), rectal (n = 4) and prostate (n = 10) cancers. There were 6 female and 14 male patients. Exclusion criteria included contraindications for MR scanning, medical implants in the pelvic area (e.g. hip prostheses) and external contour greater than the MR scanner field of view. The study was approved by a research ethics committee (reference 20/LO/0583) and all patients gave informed consent.

### 2.2 Patient imaging

The patient imaging details have been described in detail previously [16]. Briefly, patients received a planning CT scan (Sensation Open, Siemens, Erlangen, Germany) using a combined customisable foot and knee rest (Civco Medical Solutions, Coralville, Iowa, USA) with (for anal and rectal cancers) or without contrast (for prostate cancers). Patients were imaged with a comfortably full bladder and empty rectum by following appropriate bladder and bowel preparation protocols.

All patients received an MR scan on a SIGNA${}^{\mathrm{TM}}$ PET/MR 3T scanner (software version MP26 GE Healthcare, Waukesha, USA) after their radiotherapy planning CT scan and before their first treatment fraction. Patients were scanned in the radiotherapy treatment position on a flat couch-top with a coil bridge for the anterior MR coil [17] using the same model of foot and knee rest. External lasers were used for alignment by matching to patient tattoos. Patients followed the same bladder and bowel preparation protocols.

A three-dimensional (3D) radial Zero Echo Time (ZTE) sequence [18] was acquired for sCT generation. Sequence parameters were: flip angle 1°, receive bandwidth $694\;\mathrm{Hz}{\mathrm{pixel}}^{-1}$, nominal field of view $360\times 360\times 300\;{\mathrm{mm}}^{3}$, resolution $2.0\times 2.0\times 2.0\;{\mathrm{mm}}^{3}$, repetition time TR=1.06ms, nominal echo time TE=0.016ms, 59,392 3D centre-out radial-spokes and acquisition time 65s. Fat-water chemical shift effects were minimised using in-phase ZTE by adjusting the centre frequency between fat and water [19]. Image reconstruction was based on 3D gridding, including two-fold field of view extension to $720\times 720\times 600\;{\mathrm{mm}}^{3}$ (enabled by two-fold radial oversampling), deep learning-based de-noising and de-ringing [20] and 3D geometry correction. A 3D T2-weighted turbo spin echo sequence was also acquired for contouring, with sequence parameters: receive bandwidth $658\;\mathrm{Hz}{\mathrm{pixel}}^{-1}$, field of view $380\;\times \;304\;\times \;360\;{\mathrm{mm}}^{3}$, resolution $1.0\;\times \;1.0\;\times \;2.0\;{\mathrm{mm}}^{3}$, repetition time TR=2000ms and echo time TE=148ms. The scanner geometric accuracy was tested monthly during a radiotherapy quality assurance programme [21].

### 2.3 Organ at risk contours and synthetic CT generation

The MR-based OARs were generated using separate 3D U-net deep learning models for each organ on the T2-weighted MR developed previously by GE Healthcare [4]. These were trained on images from 103 patients (with 20% used for validation). The OARs generated were bladder, bowel bag, femoral heads, penile bulb, prostate, seminal vesicles, rectum and urethra. The sCT was generated from the ZTE image using a deep learning sCT algorithm also developed previously by GE Healthcare [16]. This used a two-dimensional convolutional neural network U-net model with a bone focused loss function, as described in [22], trained with 36 radiotherapy pelvis patient images. None of the patient images in this study were used to train either model.

### 2.4 Workflows

**Fig. 1:**
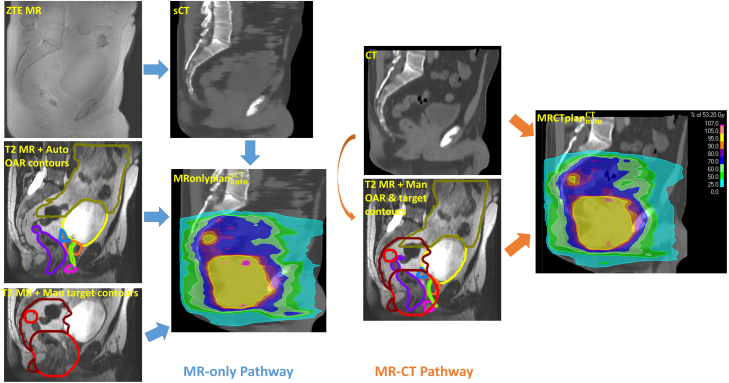
Diagrams of the MR-only workflow (left) and MR-CT workflow (right) with example images for a rectum patient. The PTVs are shown red (high dose level) and dark red (low dose level). Organs shown are bladder (yellow), bowel bag (gold), penile bulb (pink), prostate (orange), seminal vesicles (blue), rectum (purple) and urethra (green). The target contours were the same in both workflows.

The MR-only workflow consisted of automatic contours generated on the T2 MR and a sCT generated from the ZTE MR. Manual CTV contours were also produced on the T2 MR by clinical oncologists. All contours were copied over to the sCT and a plan generated (${\mathrm{MRonlyPlan}}_{\mathrm{auto}}^{\mathrm{sCT}}$). The MR-CT workflow consisted of manual OAR contours by trained medical students and reviewed by oncologists on the T2 MR and the same manual CTV contours. The planning CT was deformably registered to the T2 MR, all the contours copied over to the CT and a plan generated (${\mathrm{MRCTPlan}}_{\mathrm{man}}^{\mathrm{CT}}$). For each patient, both the MRonlyPlan and MRCTPlan were optimised by the same planner using the same beam arrangement, beam model, beam isocentre, set of optimisation objectives, objective weights and number of iterations. This sought to minimise plan variation other than due to the OAR contours (automatic and manual) and planning image (sCT and CT). Any air in the CT was outlined and over-ridden to water-density to mimic what the sCT algorithm automatically does. Images, contours and plans for both workflows are shown for an example patient in Fig. 1.

### 2.5 Workflow comparisons

Two comparisons were carried out between the automatic MR-only and manual MR-CT workflows: firstly evaluating reported dose differences between the two plans and secondly evaluating calculated dose differences between the same plan calculated on sCT and CT. The reported difference was defined as ${\mathrm{MRonlyPlan}}_{\mathrm{auto}}^{\mathrm{sCT}}$ - ${\mathrm{MRCTPlan}}_{\mathrm{man}}^{\mathrm{CT}}$. In essence, this evaluated how different the reported values from the automated MR-only workflow would be compared to the current clinical standard. The second comparison evaluated calculated differences from the MR-only plan to the manual contours on CT. This was defined as ${\mathrm{MRonlyPlan}}_{\mathrm{auto}}^{\mathrm{sCT}}$ - ${\mathrm{MRonlyPlan}}_{\mathrm{man}}^{\mathrm{CT}}$ and used a recalculation of the MRonly plan onto the planning CT. It aimed to determine what the actual OAR doses would have been delivered from the automated MR-only workflow, assuming CT and manual OAR contours were the gold standard. For both calculated and reported dose differences, mean and absolute means were calculated as a measure of the systematic and random differences respectively.

For each comparison, doses were calculated at clinically relevant OAR constraints (see Table 2). These constraints were taken from a review paper on prostate cancer [23], a stereotactic prostate radiotherapy trial [24] and the Royal College of Radiologist radiotherapy guidance for rectal [25] and anal cancers [26]. Differences in superior or inferior extent of the automatic and manual OAR contours were calculated on the T2 MR image for the bowel bag, penile bulb and rectum. The Dice similarity coefficient between the manual and automatic contours was also calculated for all OAR to facilitate comparison with other automated contouring approaches.

For a subset of five prostate patients, the automatic bowel bag contour was manually edited by an experienced consultant clinical oncologist and the time taken recorded. The measured dose difference between the edited contour and the manual contour was calculated.

## 3 Results

The mean Dice coefficients ranged from 0.38 to 0.93 (Table 1).

For the prostate patients, the mean reported dose differences were ≤1Gy; 1 percentage point for all OARs except the bowel bag (all constraints) and the femoral heads D5% (Table 2). The mean calculated dose differences were ≤1Gy; 1 percentage point for all constraints except the bowel bag D0.5 ${\mathrm{cm}}^{3}$. The mean absolute calculated dose differences were also ≤1Gy; 1 percentage point for the femoral heads, penile bulb, urethra and high dose bladder and rectum constraints. Lower dose bladder and bowel bag constraints were larger but still ≤2Gy; 2 percentage points. The bowel bag D0.5 ${\mathrm{cm}}^{3}$ and the rectum V30 Gy were >2Gy; 2 percentage points. There was a strong correlation between differences in bowel bag inferior extent between manual and automatic contours and the calculated dose difference in near maximum bowel bag dose (Fig. 2). There was also a similar correlation between differences in rectum superior extent and calculated differences in rectum V30Gy, and less strongly a correlation between differences in penile bulb superior extent and calculated dose differences to penile bulb D50% (Fig. 3).

**Table 1:** Mean ± standard error Dice similarity coefficients between automatic and manual contours for each OAR and manual contour volumes.

Organ	Mean Dice coefficient	Mean volume (${\text{cm}}^{3}$)
Bladder	0.92±0.01	169.1±23.2
Bowel bag	0.91±0.01	2959.0±198.2
Femoral head L	0.93±0.004	159.6±7.4
Femoral head R	0.93±0.005	160.3±7.5
Penile bulb^a^	0.66±0.04	6.0±1.0
Prostate^a^	0.84±0.01	45.8±5.1
Rectum	0.80±0.03	74.6±7.8
Seminal vesicles^a^	0.68±0.03	13.2±1.9
Urethra^a^	0.38±0.04	1.1±0.2

a Only for male patients (n = 14).

For the ano-rectal cohort, the mean reported and calculated dose differences were ≤1Gy for all OARs except the bowel bag D$180\;{\mathrm{cm}}^{3}$ (Table 3). The mean absolute calculated dose differences were also ≤1Gy for all constraints except the bowel bag D$180\;{\mathrm{cm}}^{3}$ and D$0.5\;{\mathrm{cm}}^{3}$. There appeared no correlation with differences in the inferior extent in the bowel bag for either D$0.5\;{\mathrm{cm}}^{3}$ or D$180\;{\mathrm{cm}}^{3}$, although there were two clear outlier results for the D$180\;{\mathrm{cm}}^{3}$ which resulted in the >1Gy mean difference (Fig. 2).

**Table 2:** Differences at clinically relevant dose constraints for the prostate cancer patients (n = 10). The square brackets in the second column indicate the units of the differences in the third and fourth column (% for differences in relative volume, Gy for differences in dose). The third column lists the mean reported differences, ${\mathrm{MRonlyPlan}}_{\mathrm{auto}}^{\mathrm{sCT}}$ - ${\mathrm{MRCTPlan}}_{\mathrm{man}}^{\mathrm{CT}}$ and the fourth the mean absolute reported difference. The fifth column gives the mean calculated differences, ${\mathrm{MRonlyPlan}}_{\mathrm{auto}}^{\mathrm{sCT}}$ - ${\mathrm{MRonlyPlan}}_{\mathrm{man}}^{\mathrm{CT}}$ and the sixth the mean absolute calculated difference. All results given as mean ± standard error.

Organ	Constraint	Reported differences		Calculated differences	
		Mean	Mean absolute	Mean	Mean absolute
Bladder	V60 Gy [%]	0.7±0.6	1.1±0.6	0.6±0.6	0.9±0.6
	V50 Gy [%]	0.9±0.5	1.3±0.3	0.8±0.6	1.5±0.4
	V40 Gy [%]	1.0±0.5	1.6±0.3	0.8±0.7	1.7±0.4
	V30 Gy [%]	1.0±0.5	1.5±0.3	0.8±0.6	1.6±0.4

	D180 ${\mathrm{cm}}^{3}$ [Gy]	−4.6±1.5	4.6±1.5	−0.2±0.3	0.7±0.2
	D100 ${\mathrm{cm}}^{3}$ [Gy]	−6.0±2.0	6.0±2.0	−0.1±0.6	1.3±0.4
	D65 ${\mathrm{cm}}^{3}$ [Gy]	−6.9±2.2	7.1±2.1	−0.3±0.8	2.0±0.5
Bowel bag	D0.5 ${\mathrm{cm}}^{3}$ [Gy]	−1.6±3.8	7.7±2.9	−2.6±4.1	7.6±3.3

Femoral head L	D50% [Gy]	0.2±0.5	1.3±0.3	−0.2±0.1	0.3±0.1
	D35% [Gy]	0.5±0.7	1.5±0.5	−0.1±0.1	0.2±0.0
	D5% [Gy]	1.1±0.6	1.8±0.4	0.0±0.1	0.1±0.0

	D50% [Gy]	0.5±0.5	1.2±0.3	−0.2±0.1	0.4±0.1
	D35% [Gy]	0.5±0.6	1.6±0.3	−0.2±0.1	0.3±0.1
Femoral head R	D5% [Gy]	1.2±0.7	2.0±0.5	0.0±0.1	0.2±0.0

Penile bulb	D50% [Gy]	0.9±0.3	1.1±0.2	0.9±0.3	0.9±0.2

	V60 Gy [%]	0.0±0.2	0.3±0.2	0.0±0.1	0.2±0.1^a^
	V57 Gy [%]	0.1±0.4	0.9±0.3	−0.1±0.3	0.8±0.2
Rectum	V30 Gy [%]	0.7±1.1	3.2±0.5	1.0±1.3	3.7±0.6

Urethra	D2% [Gy]	0.0±0.1	0.2±0.1	−0.1±0.1	0.2±0.1

a Three patients excluded because the volume receiving 60 Gy was zero.

The mean time taken to manually edit the bowel bag contour was 3.5±0.2min (3.0min, 3.7min). For this sub-cohort the mean calculated dose difference in the bowel bag D$0.5\;{\mathrm{cm}}^{3}$ improved from −4.7±8.5Gy to 1.0±3.8Gy. The mean absolute dose difference also improved from 14.6Gy to 5.1Gy.Fig. 2Plot of calculated dose constraint difference (automatic contour [sCT] - manual contour [CT]) for the bowel bag near maximum dose D0.5 ${\mathrm{cm}}^{3}$ (a) and D180 ${\mathrm{cm}}^{3}$ (b) as a function of differences in inferior extent (negative indicates automatic contour extends more inferiorly than the manual contour). Patients treated for prostate cancer are shown as blue markers and those treated for anus and rectum cancers in purple markers, with associated least squares regression lines plotted in the same colours. In (b) the dashed line indicates a least squares regression line with the two differences >1Gy excluded. Dose differences were calculated using the deformable registration. Differences in longitudinal extent measured on the T2-weighted MR image.Fig. 2

**(a):**
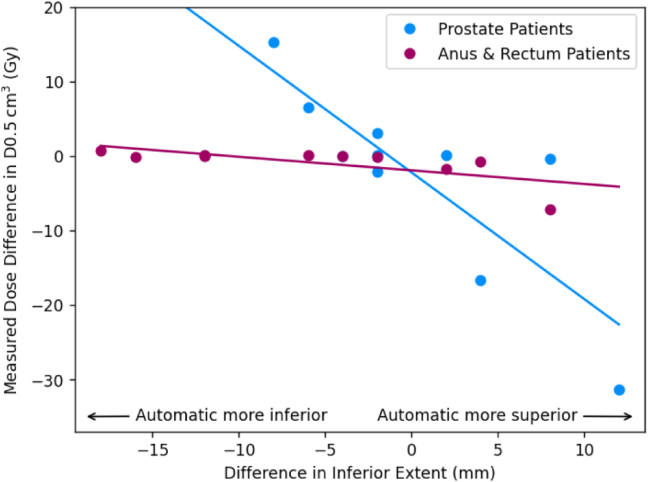
Bowel bag D0.5 ${\mathrm{cm}}^{3}$.

**(b):**
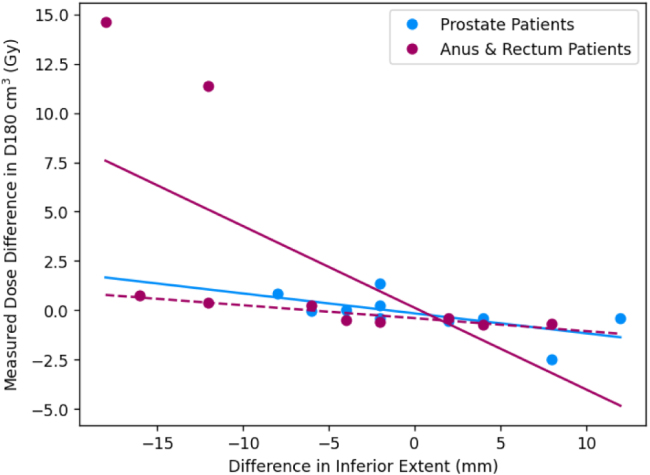
Bowel bag D180 ${\mathrm{cm}}^{3}$.

**Table 3:** Dose differences at clinically relevant dose constraints for the anal (n = 6) and rectum (n = 4) cancer patients. The third column lists the reported mean differences, ${\mathrm{MRonlyPlan}}_{\mathrm{auto}}^{\mathrm{sCT}}$ - ${\mathrm{MRCTPlan}}_{\mathrm{man}}^{\mathrm{CT}}$. The fourth column gives the reported mean absolute differences. The fifth column reports the mean calculated differences, ${\mathrm{MRonlyPlan}}_{\mathrm{auto}}^{\mathrm{sCT}}$ - ${\mathrm{MRonlyPlan}}_{\mathrm{man}}^{\mathrm{CT}}$. The sixth column gives the mean absolute calculated differences. All results given as mean ± standard error.

Organ	Constraint	Reported differences [Gy]		Calculated differences [Gy]	
		Mean	Mean absolute	Mean	Mean absolute
Bladder	D50%	0.0±0.2	0.5±0.1	−0.1±0.1	0.3±0.1
	D35%	0.1±0.2	0.6±0.1	−0.1±0.2	0.4±0.1
	D5%	0.1±0.2	0.4±0.1	−0.0±0.1	0.3±0.1

	D180 ${\mathrm{cm}}^{3}$	1.5±1.2	1.8±1.2	2.5±1.8	3.0±1.7
	D100 ${\mathrm{cm}}^{3}$	0.2±0.2	0.5±0.2	0.4±0.5	0.9±0.5
	D65 ${\mathrm{cm}}^{3}$	0.1±0.3	0.6±0.2	0.1±0.4	0.8±0.3
Bowel bag	D0.5 ${\mathrm{cm}}^{3}$	−0.8±0.9	1.4±0.8	−0.9±0.7	1.1±0.7

Femoral head L	D50%	−0.1±0.6	1.2±0.5	0.2±0.1	0.3±0.1
	D35%	−0.1±0.7	1.4±0.5	0.2±0.2	0.4±0.2
	D5%	−0.4±0.3	0.7±0.2	−0.0±0.1	0.3±0.0

	D50%	−0.3±0.5	1.2±0.4	0.0±0.0	0.1±0.0
	D35%	−0.2±0.3	0.8±0.2	0.1±0.1	0.3±0.0
Femoral head R	D5%	0.3±0.2	0.6±0.1	0.2±0.1	0.3±0.1

Penile bulb^a^	D50%	−0.2±0.3	0.5±0.1	0.0±0.3	0.4±0.1

	D95%	−0.3±0.7	1.1±0.4	−0.0±0.5	0.9±0.1
	D50%	−0.2±0.2	0.3±0.1	−0.1±0.1	0.2±0.1
Prostate^a^	D5%	−0.5±0.3	0.5±0.3	−0.4±0.2	0.4±0.2

Seminal vesicles^a^	D95%	−0.1±0.4	0.5±0.2	−0.3±0.2	0.3±0.2
	D50%	−0.2±0.1	0.2±0.1	0.0±0.3	0.4±0.2
	D5%	0.5±0.6	0.6±0.5	0.1±0.2	0.2±0.1

Urethra^a^	D2%	−0.1±0.2	0.3±0.1	0.1±0.1	0.2±0.1

a Only for male patients (n = 4).

Fig. 3Plot of calculated dose constraint difference (automatic contour [sCT] - manual contour [CT]) for the median dose to the penile bulb (D50%, a) and volume of rectum receiving lower doses (V30 Gy, b) as a function of differences in superior extent (negative indicates automatic contour extends more inferiorly than the manual contour). The associated least squares regressions lines are also plotted. Only patients treated for prostate cancer are shown. Dose differences were calculated using the deformable registration. Differences in longitudinal extent measured on the T2-weighted MR image.Fig. 3

**(a):**
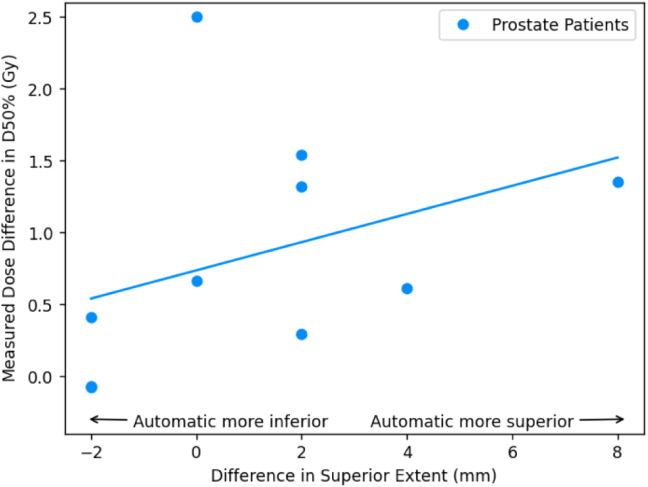
Penile bulb.

**(b):**
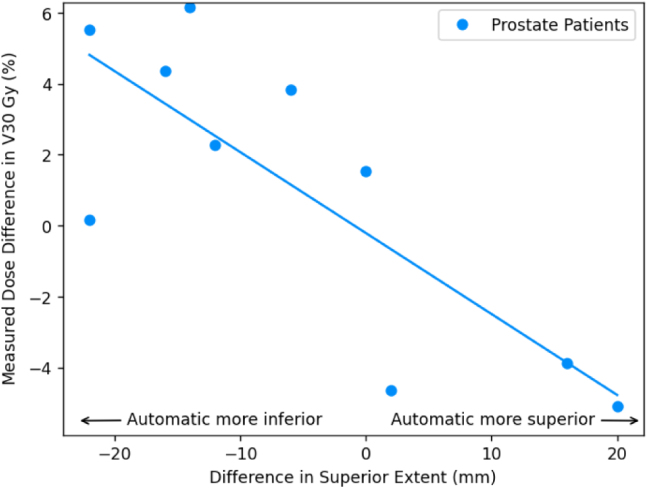
Rectum.

## 4 Discussion

This study has evaluated a combined automatic OAR contouring and MR-only workflow by comparing to the current clinical standard of manual MR contouring and MR-CT registration workflow for both prostate and ano-rectal radiotherapy patients. For prostate patients differences in mean calculated doses were ≤1Gy; 1 percentage point for all OARs except the bowel bag. Mean absolute calculated dose differences were ≤1Gy; 1 percentage point for femoral heads, penile bulb, urethra and high bladder and rectum doses (V57Gy and V60Gy). For rectum patients mean and mean absolute calculated doses were ≤1Gy for all OARs except the bowel bag.

For the prostate patients, differences in reported doses at clinically relevant dose constraints were ≤1Gy; 1 percentage point for all OARs except the bowel bag and the femoral head D5%. This compared the doses reported from the automatic MR-only workflow (${\mathrm{MRonlyPlan}}_{\mathrm{auto}}^{\mathrm{sCT}}$) with those reported from the current clinical standard (${\mathrm{MRCTPlan}}_{\mathrm{man}}^{\mathrm{CT}}$). In an automatic MR-only workflow, these reported doses would be all that was available, and it was important that these differences were small. This was defined as ≤1Gy; 1 percentage point in relative volume based on data showing that inter-observer variability in manual contouring resulted in dose differences of 1Gy [27]. Therefore for the bladder, femoral heads (D50% and D35%), penile bulb, rectum and urethra, the combined automatic MR-only pathway results in similar dose differences as those that occur between manual users (the current clinical standard).

A previous evaluation of the dose calculation accuracy of the sCT gave dose differences of ≤0.5% (≤0.3Gy) [16]. This implies that the majority of the differences were due to differences between automatic and manual contours, rather than sCT dose calculation inaccuracies. The bowel bag had large differences for all constraints (Table 2), which suggests that it is not sufficiently accurate to be used without editing. This fits with it being given the lowest qualitative rating by clinicians [4]. Differences in femoral head D5% were more unexpected given the very high clinician rating [4], but visual inspection of the dose distributions showed that the MRonlyPlan and MRCTPlan could be quite different in the region of the femoral heads. This was likely due to femoral head doses for all plans being a long way below constraints, and may reflect differences in where the optimiser was pushing lower doses between the two plans due to differences in other OARs, rather than differences in the femoral heads. This fits with calculated dose differences (comparing doses from the same plan), which were ≤0.2% for the femoral heads.

The calculated dose differences were also ≤1Gy; 1 percentage point for all OARs except the bowel bag D$0.5\;{\mathrm{cm}}^{3}$. This tested what a plan produced using the combined automatic MR-only workflow (${\mathrm{MRonlyPlan}}_{\mathrm{auto}}^{\mathrm{sCT}}$) would have actually delivered, using the manual MR contours and CT scan as a gold standard (${\mathrm{MRonlyPlan}}_{\mathrm{man}}^{\mathrm{CT}}$). This suggests the combined automatic workflow has similar systematic differences as different manual observers would have. However it is also important to consider the random errors, indicated by the mean absolute differences. These were also ≤1Gy for the femoral heads, penile bulb and urethra, suggesting they perform as least as well as manual observers do. For the bladder the high dose volume difference (V60Gy) was <1 percentage point, although the lower dose volumes were only ≤1.7 percentage points. It is harder to interpret these differences since Vaassen et al. only reported mean and not mean absolute dose differences between manual contours drawn by different radiation therapists [27], therefore the ≤1 percentage point limit may not be applicable for random errors. This does imply that discrepancies in bladder volume were likely to occur in regions receiving lower doses.

There was a similar situation with the mean absolute calculated differences in the rectum. The high dose volume differences (V60Gy and V57Gy) were <1 percentage point, although the V30Gy was much larger (3.7%). However, there was a strong correlation in calculated dose differences in the rectum V30Gy and differences between manual and automatic contours in the rectum superior extent (Fig. 3). This implies that near the prostate PTV, the manual and automatic rectum contours were very similar (hence very small differences in volumes receiving high doses), but differences in superior extent of 10–20 mm would increase the overall volume of the rectum receiving zero dose, and so bring down the relative volume of the rectum receiving 30 Gy. Defining the superior border of the rectum is a known issue within contouring for prostate radiotherapy, with significant variability reported in manual contouring [28]. This suggests these differences may be similar to inter-observer variability in manual contours.

The calculated differences in bowel bag D$0.5\;{\mathrm{cm}}^{3}$ were strongly correlated with differences between automatic and manual contours in the inferior extent of the bowel bag. This was because for prostate radiotherapy there is a steep dose gradient in the inferior-superior direction and so differences of a few slices (5–10 mm) could result in differences of 10–15 Gy (Fig. 2). For the five patient sub-cohort with the automatic bowel bag subsequently edited, the mean calculated dose difference in the bowel bag D$0.5\;{\mathrm{cm}}^{3}$ improved from −4.7±8.5Gy to 1.0±3.8Gy and the mean absolute dose difference from 14.6Gy to 5.1Gy. These manual edits only took 3.5 min on average, suggesting it was a relatively minor edit. The mean absolute dose difference was still 5.1Gy, indicating the large dose differences possible even between two manual contours in the presence of a steep dose gradient.

For the ano-rectal patients, differences in both reported and calculated doses were ≤1Gy; 1 percentage point for all OARs except the bowel bag D$180\;{\mathrm{cm}}^{3}$. Again this suggests that the combined automatic MR-only workflow performs as well as the manual MR-CT workflow for all OARs except the bowel bag. Mean absolute calculated dose differences were also ≤1Gy for all constraints, suggesting random errors were also small for this cohort. Interestingly both measured and calculated dose differences for the bowel bag were ≤1Gy for all constraints except the D$180\;{\mathrm{cm}}^{3}$, unlike for the prostate patients where reported doses were different for all constraints. This was the case even though there were the same range of differences between automatic and manual contours in inferior extent for the ano-rectal patients as the prostate patients (Fig. 2). This is likely due to the fact that there was a much flatter dose distribution overlapping the bowel bag for ano-rectal patients compared to prostate patients (see Fig. 1 for an example dose distribution of a rectum patient). This highlights that although dose differences are the most relevant method of evaluating contours [8], it is also highly dependent on the dose distribution used for the evaluation. Therefore, automatic contours assessed as clinically suitable for one clinical site may not be acceptable for other treatment sites. This can also be seen in the discrepancies between mean Dice coefficients and dose differences for some of the OARs, such as the penile bulb and urethra, which had relatively low Dice coefficients but small dose differences. This is likely due to these being small structures with Dice coefficient being strongly correlated with volume [29]. This means small deviations in these small OARs generated larger reductions in Dice coefficient than for larger OARs such as bladder or rectum (see Table 1), even though they did not result in significant dose differences.

The calculated differences in bowel bag D$180\;{\mathrm{cm}}^{3}$ did not have any direct correlation on differences in inferior extent, but there were two outlier patients with large measured dose differences, who also had large differences in inferior extent (Fig. 2). Investigation of these outlier patients showed that at the level of the difference in inferior extent there was a nodal PTV volume where the dose was boosted to 50.4Gy. The difference in inferior extent between automatic and manual contour meant a significant change in how much this boosted dose region was encompassed by the bowel bag, and therefore a large difference in the bowel bag D$180\;{\mathrm{cm}}^{3}$. The other two patients with similar differences in inferior extent of the bowel bag did not have a nodal PTV and therefore no difference in the bowel bag D$180\;{\mathrm{cm}}^{3}$.

Combined MR-based automatic contouring and MR-only radiotherapy workflows for the pelvis have not been reported previously. Dice coefficients for MR-based deep learning OAR models in the literature were similar to those found in this study. Elguidini et al. evaluated a deep learning MR-based OAR contouring model on 50 prostate radiotherapy patients [6]. They reported Dice coefficients that were within 2× the standard error of the results reported here for the bladder, prostate, penile bulb and rectum. Their reported value for the urethra (0.69±0.10) was substantially higher than found in this study. Sha et al. found dice coefficients of 0.93 for bladder and both femoral heads for a U-NET deep learning MR model for rectum radiotherapy patients [7]. These agree within 0.01 of the results found in this study. Vaassen et al. calculated dose differences between deep learning MR-based automatic contours and manual contours for 76 brain radiotherapy patients [30]. Median dose differences were within ±1Gy for all eight OARs, despite median Dice coefficients ranging from 0.43 to 0.92. This is similar to results found in this study, where for the smallest OAR (urethra) the mean dose difference was only 0.1Gy despite a mean Dice coefficient of 0.38.

This study had some limitations. Principally, there were a limited number of patients investigated, particularly in the ano-rectal cohort given the variation in prescribed doses and presence and location of nodal PTVs. This suggests that it is difficult to generalise from the results of this study to all ano-rectal patients, especially given the strong dependence on dose differences between automatic and manual contours on the specifics of the dose distribution reported in this study. Further work could investigate more patients to further characterise the accuracy of the automatic MR-only workflow for ano-rectal radiotherapy. Another limitation of this study was the fact that the sCT algorithm was trained on data acquired from the same scanner with the same imaging protocol. It would be important to test the generalisation of these algorithms to data acquired on different scanners and with varying imaging protocols. Finally, this study has used dose differences to evaluate the automated workflow. These are clinically relevant criteria, but may not capture all the underlying differences between the automated and manual workflows.

In conclusion, a combined automatic OAR contouring and MR-only workflow has demonstrated mean calculated differences to a manual OAR contouring and MR-CT fusion workflow that were ≤1Gy; 1 percentage point for all OARs except the bowel bag for both prostate and ano-rectal radiotherapy patients. Mean absolute differences were also ≤1Gy; 1 percentage point except for bowel bag and bladder and rectum low dose volumes (prostate patients only). This suggests that the automatic MR-only workflow showed similar dose differences to those arising from manual inter-observer variability, except for those OARs. All automatic OAR contours would still require careful per-patient review and editing prior to clinical use. This warrants further evaluation of the combined automatic OAR MR-only workflow with patients from a range of pelvic radiotherapy sites.

## CRediT authorship contribution statement

**Jonathan J. Wyatt:** Conceptualization, Methodology, Investigation, Writing – original draft. **Sandeep Kaushik:** Software, Writing – review & editing. **Cristina Cozzini:** Software, Writing – review & editing. **Bernadett Kolozsvári:** Software, Writing – review & editing. **Borbála Deák-Karancsi:** Software, Writing – review & editing. **Rachel A. Pearson:** Writing – review & editing. **Steven Petit:** Conceptualization, Funding acquisition, Writing – review & editing. **Marta Capala:** Writing – review & editing. **Juan A. Hernandez-Tamames:** Funding acquisition, Writing – review & editing. **Katalin Hideghéty:** Funding acquisition, Writing – review & editing. **Ross J. Maxwell:** Supervision, Funding acquisition. **László Ruskó:** Software, Funding acquisition, Writing – review & editing. **Florian Wiesinger:** Conceptualization, Funding acquisition, Writing – review & editing. **Hazel M. McCallum:** Conceptualization,Supervision, Funding acquisition, Writing – review & editing.

## Declaration of competing interest

The authors declare that they have no known competing financial interests or personal relationships that could have appeared to influence the work reported in this paper.
